# Cellular senescence and abdominal aortic aneurysm: From pathogenesis to therapeutics

**DOI:** 10.3389/fcvm.2022.999465

**Published:** 2022-09-14

**Authors:** Ding Wang, Xinyu Hao, Longyuan Jia, Yuchen Jing, Bo Jiang, Shijie Xin

**Affiliations:** ^1^Department of Vascular Surgery, The First Affiliated Hospital of China Medical University, Shenyang, Liaoning, China; ^2^Key Laboratory of Pathogenesis, Prevention and Therapeutics of Aortic Aneurysm, Shenyang, Liaoning, China

**Keywords:** pathogenesis, cellular senescence, therapeutics, longevity, abdominal aortic aneurysm

## Abstract

As China’s population enters the aging stage, the threat of abdominal aortic aneurysm (AAA) mainly in elderly patients is becoming more and more serious. It is of great clinical significance to study the pathogenesis of AAA and explore potential therapeutic targets. The purpose of this paper is to analyze the pathogenesis of AAA from the perspective of cellular senescence: on the basis of clear evidence of cellular senescence in aneurysm wall, we actively elucidate specific molecular and regulatory pathways, and to explore the targeted drugs related to senescence and senescent cells eliminate measures, eventually improve the health of patients with AAA and prolong the life of human beings.

## Introduction

Abdominal aortic aneurysm (AAA) is locally weak and aneurysm-like dilatation of the abdominal aorta, diameter > 3 cm or more than 1.5 times the normal diameter (1). AAA is a common disease among the elderly, and the incidence of AAA increases with age. In one study, the incidence of AAA is reported to be 55 per 100,000 person-years in males aged 65–74, 112 per 100,000 person-years in males aged 75–85, and 298 per 100,000 person-years in males older than 85 (2). Most AAA patients are asymptomatic and are discovered accidentally during a physical exam or ultrasound screening. However, rupture or precursor rupture may occur when patients present with symptoms such as lumbago and abdominal pain. The in-hospital mortality rate of rupture is about 40% (3), while the out-of-hospital mortality rate can be as high as 90% (4), resulting in about 150,000–200,000 deaths globally (5), which is a serious threat to patients’ health. Currently there is no effective method to inhibit AAA progress in the treatment of clinical drug. Usually, surgical treatment (open surgery or endovascular stent repair) is the only treatment for AAA patients whose diameter is greater than 5.5 cm (greater than 5.0 cm for women) or increases by more than 1.0 cm every year (6). Patients who fail to meet the surgical indications need long-term follow-up (7), which brings heavy economic burden and psychological pressure to patients. Therefore, finding and developing potential therapeutic targets and drugs to delay aneurysm progression or prevent AAA rupture is of great clinical significance and can significantly extend human life expectancy.

**FIGURE 1:**
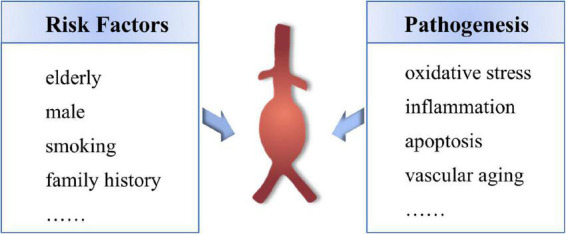
The remain risk factors and pathogenesis of AAA.

The mechanism of the occurrence and development of AAA has not been fully clarified, mainly including oxidative stress, immune inflammatory response, apoptosis of vascular smooth muscle cells (VSMCs) and vascular aging (8–10). The main risk factors for AAA are age over 65, male, smoking habit and family history (11) (Figure 1). With the progress of the population aging in China, the elderly population in 2050 is expected to appear the big bang, 65 years of age or older population will reach 400 million (accounts for 26.9% of the total population), more than 80-year-old aging population will reach 150 million (12), China will become one of the countries with the highest proportion of elderly people in the world, and which will inevitably cause a series of social and economic challenges (13). Therefore, it is important to elucidate the specific mechanisms involved in the pathogenesis of age-related AAA, and to provide potential therapeutic targets for the development of novel therapeutic agents that inhibit the expansion and rupture of AAA.

Hayflick and Moorhead were the first to describe “Replicative senescence (RS)” in 1961, which referred to the limited ability of human cells to proliferate (cell cycle arrest). Senescence is the irreversible loss and depletion of the cell’s ability to differentiate and divide (14). The “telomere hypothesis” emerged in the 1890s as one of the most important molecular mechanisms of RS, generating much scientific interest in the study of senescence (15). Later, it was proved that senescence may be caused by other genetic damage, such as DNA damage, chromosomal aberrations and chromatin aggregation (16, 17). Now, it is recognized that senescence also acts as a defense mechanism in response to various stresses, including telomere wear, oncogene activation, tumor suppressor gene inactivation, oxidative stress, mitochondrial dysfunction and DNA damage (radiation, chemicals, and ROS) (18, 19). This process is classified as stress-induced premature senescence (SIPS). RS is characterized by telomere shortening. Telomeres are regions thousands of bases long in DNA, covering the ends of chromosomes, and they play an essential role in maintaining the integrity and stability of DNA molecules (20). Because DNA polymerase cannot replicate the end of the DNA molecule completely each time, the DNA molecule loses 50–200 base pairs of telomeres at the 3′ end after each replication (21). Hayflick et al. observed a limit, known as the Hayflick limit, which was the maximum number of cell divisions (about 50–70) (14). When the Hayflick limit is reached, the integrity of DNA molecules is broken and the ends of chromosomes are exposed, leading to replicative senescence (22). And SIPS is triggered by telomere-independent DNA damage responses (DDR) caused by both internal and external stressors, which is often involved in the activation of cycle-dependent kinase inhibitor (CDKI) p21 and exogenous tumor suppressors (p16 and Rb) (23). CDK mediates the transition among different stages of the cell cycle, which stagnates when interrupted by DNA damage. Once stopped, the cells face three outcomes: after DNA damage is repaired, cells can re-enter the cell cycle; If not, cells die and are cleared; If still in stasis, they become senescent cells. Thus, cell cycle arrest is the first step of senescence (24).

## Hallmarks of cellular senescence

**FIGURE 2:**
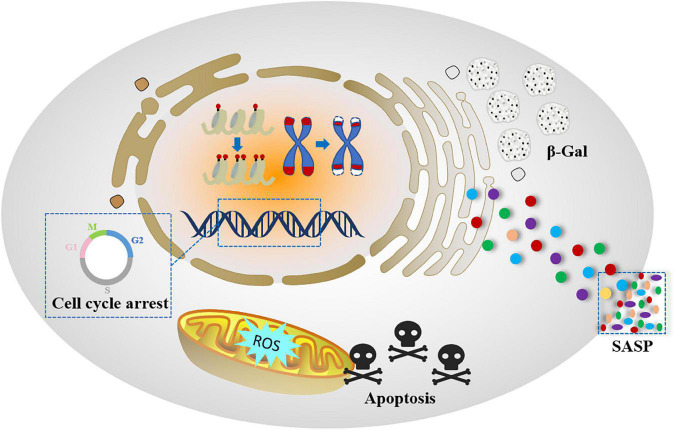
Pattern diagram of senescent cell hallmarks.

Although there is no consensus on the senescence process and related mechanisms, the scientific community has proposed a senescent cell phenotype that is characterized by specific changes during cellular senescence occurred. In addition to cell cycle arrest, senescent cells undergo morphological, biochemical and functional changes, which are signs of cellular senescence (25) (Figure 2).

### Morphogenesis and chromatin remodeling

Senescent cells undergo significant morphological and structural changes, including enlargement, flattening, nuclear enlargement, multinucleation (26) and changing the composition of plasma membrane (27, 28). These seemingly simple changes help establish and maintain the senescent state of cells.

Senescent cells have also been observed with significant chromatin related changes, most notably the formation of senescence associated heterochromia lesions (SAHF), which were first described by Lowe et al. (29). SAHF, as a DNase-resistant and DAPI-dense subnuclear cell structure, enriches in histone modification (H3K9me) associated with transcription inhibition (30). The formation of SAHF leads to transcriptional inhibition of E2F target genes, which triggers permanent cell cycle arrest (31). The emergence and function of SAHF depend on an effective INK4A-Rb pathway, since INK4A inactivation prevents SAHF formation and Rb is recruited into proliferation-related genes to inhibit them (32). However, recent research results challenged some of earlier explanations. It has been reported that cellular senescence can be robustly established in the absence of SAHF. One study found that telomere shortening, ionizing radiation, and prolonged exposure to hydroxyurea or etoposide also can trigger senescence without the distinct formation or the typical heterochromatin markers of SAHF (33). Surprisingly, SAHF formation was observed under similar conditions but with different cell lines, which may be related to INK4A induction (31).

### Senescence-associated-β galactosidase (SA-β-Gal)

Detection of SA-β-Gal activity is the most commonly used senescence discrimination method (27). Endogenous β-Gal is the product of GLB1 gene encoding (34), and the transcription process is negatively regulated by the NOTCH1 signaling pathway (35). The lysosome content in senescent cells increases significantly, leading to a significant increase in the endogenous β-Gal activity of lysosomes (34). Lysosomal β-galactosidase activity is usually most active at pH 4.0, but which can also be detected at pH 6.0 in senescent cells (36). Therefore, SA-β-Gal activity can reflect lysosome β -galactosidase activity, thus revealing the increase of lysosome content in senescent cells. Although these histochemical and immunohistochemical staining methods are widely performed, SA-β-Gal activity is not only present in senescent cells (37). Studies have found that cells lacking GLB1 gene undergo unimpeded senescence (38). In addition, osteoclasts and macrophages themselves have higher levels of β-Gal activity (39, 40). Therefore, SA-β-Gal activity assay combined with other markers may be more reliable for determining cellular senescence.

### Senescence-associated secretory phenotype

Senescent cells influence surrounding environment and communicate with their neighbors by producing a complex mixture of secreted factors that change the behavior of both themselves and nearby non-senescent cells (41–43).

The attribute of senescent cells secreting pro-inflammatory cytokines, chemokines, angiogenic factors, and proteases is referred to as senescence-associated secretory phenotype (SASP) (44). SASP can have positive or negative effects on the organism. When it is excessively elevated or persistent, SASP causes local and potentially systemic inflammation, destroys tissue structure, and stimulates adjacent cells to senescence. In contrast, local or transient SASP stimulation may promote recovery of tissue damage (44). Cytokines IL-6 and IL-8 in SASP enhance the growth stagnation of senescent cells, which is detrimental (45). MMPs, the component of SASP, can limit fibrosis after liver injury or during skin wound healing, which is beneficial (46, 47). SASP enhances and spreads senescence through autocrine and paracrine. IL-1α, TGF-β and IL-6 promote senescence in a cellular autonomous manner, while many other SASP factors act in a non-cellular autonomous manner, thus altering the behavior of neighboring cells (48, 49).

SASP is highly heterogeneous and regulated at multiple levels (50). Transcriptional level regulation: SASP is mainly regulated by activation of nuclear factor kappa-B (NF-κB) and CCAAT/enhancer binding protein β (C/EBPβ) transcription factors (47). Janus kinase (JAK), transcription activator (STAT) pathways and NOTCH signaling are also involved in regulating SASP expression (51). Epigenetic regulation: Bromine domain and extra-terminal (BET) family protein BRD4 recruited the super enhancer element adjacent to SASP gene, resulting in the remodeling of the enhancer (52). Decreased histone H3K9me2 (53) and histone deacetylase silencing information regulator 2 related enzyme 1 (SIRT1) (54), increased expression of histone variant macroH2A1 (55) in the promoter region of key SASP factors are involved in the regulation of SASP. Histone variant H2AJ accumulated in senescent cells is also involved in the induction of SASP (56). Other epigenetic mediators, such as histone lysine-n-methyltransferase 2A (MLL1 and HMGB2), also promote SASP production by keeping their gene loci open and active (57, 58). Post-transcriptional regulation: Rapamycin (mTOR) is a key regulator of protein translation in senescent cells. MTOR mainly regulates SASP through two mechanisms. On the one hand, mTOR promotes IL-1α translation and activates NF-κB and C/EBPβ (59). On the other hand, mTOR indirectly inhibits the RNA-binding protein ZFP36L1, preventing it from degrading the mRNA encoding SASP factor (60).

### P53/p21 and p16/Rb pathways

The p53/p21 signaling pathway is activated in response to DNA damage caused by oncogenic factor mutations, telomere wear or oxidative stress, thereby inducing cellular senescence (61, 62). The activation of p53 is regulated by post-translational modifications, including ubiquitination, methylation, phosphorylation and acetylation (63). Mouse double minute 2 (MDM2) is an E3 ubiquitin ligase that plays an important role in p53 degradation as a negative regulator (64). Activated p53 regulates the expression of numerous anti-proliferation genes and participates in cellular senescence (65, 66). In addition, p53 is an important tumor suppressor protein that regulates p21 transcription process (67). P21 is a cyclin kinase (CDK) inhibitor that inactivates all CDKs, thereby inhibiting cell cycle progression. Specifically, p21 inhibits the CDK complex activity by interacting with two cyclin-binding mods (Cy1 and Cy2), resulting in the removal of phosphorylation of the Rb protein family and subsequent binding to E2F to form the DREAM complex, eventually which leads to cell cycle stagnation (68, 69). P21 also promotes senescence by down-regulating mitotic gene cyclin E2 and up-regulating senescence-related gene fibronectin-1 (70). However, p21 can also be activated in a p53-independent manner, induced by pathway such as TGF-β and by using Sp1 as a main transcription factor (71–73).

P14 (ARF), p15 and p16 are three tumor suppressor proteins encoded by INK4/ARF gene (74). ARF regulates the stability of p53 by binding to MDM2, whereas p53 regulates the expression of ARF through a negative feedback mechanism (75, 76). P16 directly binds to CDK4/6 and blocks the formation of CDK4/6 complex, thereby inhibiting Rb phosphorylation and facilitating E2F target gene expression (77). Rb family is one of the main targets of CDK complex, and its most significant function is to bind and inactivate E2F complex and prevent transcription of E2F target genes. Dephosphorylated pRb binds to E2F to form the Rb-E2F complex that inhibits transcription of genes involved in cell cycle progression by binding to the promoter region of E2F target genes (78). This inhibitory mechanism is eliminated by CDK2-mediated Rb hyperphosphorylation, which releases E2F and promotes S-phase gene transcription and cell cycle progression (79). In addition, p16 has also been reported to induce cellular senescence in a manner independent of p53 (80).

### Apoptosis resistance

Apoptosis is a kind of spontaneous and orderly death controlled by genes, which helps to maintain homeostasis (81, 82). Various pro-apoptotic signals initially stimulate different signaling pathways and eventually converge into a common mechanism: up-regulating the expression of cysteine proteases, activating caspase and executioner caspase, and which ultimately leads to degradation of cell components (83). This mechanism is negatively regulated by a variety of genes, the most common of which is the Bcl-2 family (84). Studies have shown that anti-apoptotic factors Bcl-W, Bcl-XL and Bcl-2 of the Bcl-2 family are significantly upregulated in senescence induced cells, and these proteins can successfully induce cellular senescence after being removed (85, 86). However, there are still great differences in apoptosis resistance among different cell types (87).

### Metabolic reprogramming

Senescent cells undergo a series of metabolic changes related to increased oxidative stress and oxidative protein accumulation, impaired protein homeostasis and specific metabolic pathways (88). Increased metabolic activity is considered to be an adaptive change, and senescent cells need metabolic reprogramming to meet their nutritional and energy requirements in the senescent state, such as increased SASP secretion, oxidative stress and endoplasmic reticulum stress (87). In addition, studies have also found that autophagy in senescent cells is combined with protein synthesis to overcome the amino acid shortage (89). Meanwhile, the combination of metabolomics and proteomics has revealed that senescent cells can flexibly use different metabolic pathways to improve survival and support function. Specifically, the tricarboxylic acid (TCA) cycle, pentose phosphate and nucleotide pathways are upregulated in senescent cells, while the fatty acid (FA) pathway is downregulated (90).

## Relationship between cellular senescence and abdominal aortic aneurysm

AAA is a degenerative cardiovascular disease, partly characterized by local weakness of the abdominal aorta, which may be an objective manifestation of changes in the internal microenvironment of local cellular components and extracellular matrix components, namely, the result of vascular remodeling. In the process of vascular remodeling, the most significant feature is the loss of VSMCs and infiltration of immune inflammation, which is very consistent with the two typical characteristics of senescent cells, the reduced proliferation ability and SASP, suggesting that cellular senescence may play an important role in the pathogenesis of AAA. It can’t be ignored that AAA is more common in elderly patients, and elderly patients are the main population of aging. Below, we will elaborate on the evidence and mechanisms of local and circulatory senescence in patients with AAA (Table 1).

**TABLE 1:** Evidence of cell senescence in the aneurysm wall.

Sample types	Sample sources	Senescent signs	References
Vascular SMCs	AAA tissues	Larger and rounder in appearance, reduced proliferation ability and limited *in vitro* life.	(101)
	IMA tissues		
Vascular SMCs	AAA tissues	Prominent “rhomboid” morphology, increased spread area, impaired proliferation and SA-β-gal activity.	(102)
	Saphenous vein tissues		
Vascular ECs	AAA tissues	Decreased telomerase expression.	(103)
	Normal aorta tissues		
Circulating leucocytes	AAAs group	Reduced telomere length.	(104, 105)
	Normal aortas group		
Vascular MSCs	AAA tissues	Decreased proliferation and migration ability, increased mitochondrial fusion, ROS production and SA-β-gal activity,	(106)
	Normal aorta tissues	Decreased mitochondrial membrane potential and autophagy level, downregulation of IL-10 secretion, upregulation of IL-6 and TNF-α secretion.	
Vascular MSCs	AAA tissues	Decreased proliferation, increased cell surface area and ROS production, activation of the p21, p16 and DNA damage response, dysregulated autophagy.	(107)
	Normal aorta tissues		
Circulating EPCs	AAAs group	Increased SA-β-gal activity.	(108)
	Normal aortas group		

## Evidence and mechanisms of cellular senescence in abdominal aortic aneurysm

A large number of studies have found that the formation and progress of AAA is closely related to cellular senescence. Liao et al. obtained VSMCs derived from paired AAA and adjacent (non-aneurysmal) inferior mesenteric artery (IMA) using explants and observed their differences during successive passages in culture. The results showed that VSMCs derived from AAA were larger and rounder in appearance than VSMCs derived from IMA, with significantly reduced proliferation ability and limited *in vitro* life (91). This is the first report of signs of senescence in AAA medial VSMCs, which lays the foundation for us to explore the correlation between the two. VSMCs senescence characterization has also been confirmed in human end-stage aneurysm tissues and age-matched saphenous vein tissues (92). Subsequent study has shown that, compared with the control group, the expression of telomerase in endothelial cells of AAA patients is significantly reduced. The association persisted after adjusting for age, sex, coronary artery disease, hypercholesterolemia, hypertension and smoking (93). The finding further suggests that cellular senescence is closely related to AAA, and we have reason to believe that cellular senescence is a key link in the pathogenesis of AAA. Meanwhile, it has been reported that circulating leukocyte DNA content can predict vascular telomere content, which can serve as an accurate proxy for human vascular age (94, 95). In recent years, it has been found that the mesenchymal stem cells (MSC) of aneurysm wall have senescence state, which promotes the occurrence and development of AAA. Compared with adipose-derived mesenchymal stem cells from healthy donors (H-ASC), adipose-derived mesenchymal stem cells from patients with AAA (AAA-ASC) were characterized by enhanced senescence, such as increased SA-β-Gal activity and decreased proliferation and migration. In addition, AAA-ASC showed decreased cell function with mitochondrial kinetic disorder, production of reactive oxygen species (ROS) and decreased mitochondrial membrane potential (96). Similar studies have found that vascular-resident mesenchymal stromal cells (AAA-MSC) and circulating endothelial progenitor cells (EPCs) in patients with AAA have the characteristics of senescent cells and damage of vascular repair ability of MSC (97, 98). Besides, another study has found that pretreatment of elderly mice with oral anti-aging agents (dasatinib + quercetin) can reduce the abundance of senescent cells in the arterial wall and surrounding tissues, and inhibit the severity of Ang II-induced AAA (99). Further elucidation that cellular senescence supports the occurrence and development of AAA, and reversal of cellular senescence is helpful to explore a new strategy to restore AAA therapy.

VSMCs play a key role in maintaining vascular system development and normal vascular homeostasis (100–102). Under physiological conditions, VSMCs are located in the vascular media, and their main functions are to regulate vasoconstriction, blood pressure, arterial tension diameter and flow distribution (103). VSMCs have differentiated plasticity in response to abnormal environmental stimuli, and tend to transform from a contractile phenotype to a synthetic phenotype and secrete pathogenic factors, such as ROS, inflammatory cytokines and MMPs (104). Importantly, oxidative stress and the secretion of inflammatory cytokines are also important features of cellular senescence. The phenotypic transformation process has been fully verified and is closely related to the occurrence and development of AAA (105). In terms of mechanism, cellular senescence is also an important pathological process in the formation of AAA. In addition, aging has become an independent risk factor for cardiovascular diseases (CVDs), so how cellular senescence is involved in CVDs has been the focus of researchers for the past several decades (106). Signs of VSMCs senescence have been found in aging vascular walls, represented by reduced proliferation rate and phenotypic transformation (107). Although the current understanding of VSMCs senescence is not fully adequate, it is necessary to decipher the correlation between senescence-related molecules and AAA progression in the search for new therapies.

### SIRT1-related senescence and abdominal aortic aneurysm

The SIRT protein family is a group of histone deacetyltransferases with nicotinamide adenine dinucleotide (NAD)^+^ dependence, including seven homologous genes of SIRT1-SIRT7 (108). SIRT1 is the most well-studied member of the SIRT protein family and has been proven to regulate senescence and age-related diseases (109, 110). A Study has shown that endogenous SIRT1 expression in VSMCs of elderly donors is significantly reduced compared with that of young donors. The loss of SIRT1 expression leads to cellular senescence in VSMCs, which is related to cell proliferation and migration ability, even VSMCs from young donors showed cellular senescence after si-SIRT1 knockout (111). These data suggest that down-regulation of SIRT1 motivates to VSMCs senescence. It is found that the expression and activity of SIRT1 are significantly reduced in human AAA samples. VSMC-specific knockout of SIRT1 accelerates Ang II-induced AAA formation and rupture and AAA-related pathological changes, while VSMC-specific overexpression of SIRT1 inhibits Ang II-induced AAA formation and progression. Mechanistically, SIRT1 inhibits p21-dependent VSMCs senescence by blocking Ang II-induced binding of NF-κB to monocyte chemotactic protein-1 promoter (MCP-1) (112). In addition, the role of phosphodiesterase 1C (PDE1C) in VSMCs senescence *in vitro* and *in vivo* depends on SIRT1. Mechanism studies further demonstrate that inhibiting cAMP by PDE1C stimulates SIRT1 activation, which leads to subsequent upregulation of SIRT1 expression. It is also found that the pharmacological inhibition of PDE1C significantly slows down the progression of AAA (113). Importantly, SIRT1 activators show good effects in regulating lipid metabolism, inhibiting inflammation and protecting CVDs (110).

Non-coding RNAs play an important role in regulating the formation of AAA and the senescence of VSMCs (114–116). Upregulation of miR-199a-5p, enhanced expression of p-p53 and p21, and decreased expression of SIRT1 were observed in plasma and Ang II-treated VSMCs of patients with AAA. Bioinformatics prediction of TargetScan database and analysis of dual luciferase reporter genes showed that the 3′-UTR of SIRT1 had a potential binding site for miR-199a-5p, indicating that SIRT1 was a potential target of miR-199a-5p. Mechanically speaking, Ang II treatment significantly increased the level of miR-199a-5p, thereby promoting VSMCs senescence to participate in the formation of AAA by inhibiting the expression of SIRT1 (117). During the continuous culture of human aortic VSMCs *in vitro*, an increase in miR-34a level was observed accompanied by down-regulation of SIRT1. Surprisingly, even in young human aortic VSMCs, miR-34a overexpression and SIRT1 inhibition may induce cellular senescence. However, cellular senescence induced by overexpression of miR-34a in VSMCs was saved by transfection of SIRT1 protein, implying that the pro-senescence effect of miR-34a depended on SIRT1 participation (118). In addition, a research has confirmed that miR-34a induces senescence in endothelial cells, which is also achieved by targeted inhibition of SIRT1 (119). Meanwhile, other study has found that miR-188-5p with senescence induction in aneurysm wall co-locates with CD68 and CD3, suggesting that miR-188-5p may regulate the senescence of macrophages and T cells to involve in the occurrence and development of AAA (120). Therefore, different cell types may have different mechanisms of senescence regulation. In addition to miRNAs, long non-coding RNAs (lncRNAs) are also participated in AAA lesions and VSMCs senescence. Both ANRIL and SIRT1 were down-regulated and miR-181a expression was up-regulated in dual-channel-triggered senescent VSMCs. However, the overexpression of ANRIL prominently restrained senescence and promoted VSMCs viability *via* p53/p21 regulatory pathway. Mechanically, ANRIL overexpression inhibited miR-181a expression, which decreased the 3′-UTR inhibitory targeted binding of miR-181a to SIRT1 and increased SIRT1 expression (120). In the IgE-induced VSMCs senescence model, lincRNA-p21 was found to be upregulated, thereby enhancing p21 expression without altering p53 expression. Rescue experiments also proved that IgE induced VSMCs senescence and aggravated the development of AAA by upregulation of lincRNAp21/p21 pathway (121). The above studies provide more new potential targets for the treatment of AAA.

**FIGURE 3:**
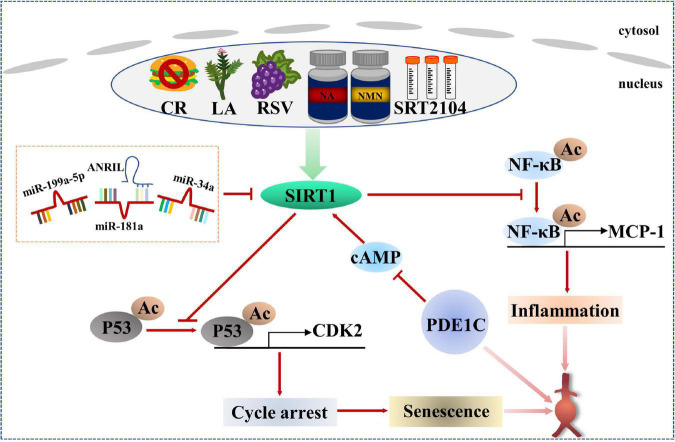
Regulation mechanism and intervention between SIRT1-related senescence and AAA.

Therefore, it can be seen that SIRT1 is a key factor in regulating cellular senescence, and it must be the most promising protective regulator for AAA. It was found that the protective effect of calorie restriction (CR) on AAA development was eliminated in VSMC-specific SIRT1 knockout mice (122). In addition, licorice chaltone A (LA), an ingredient in licorice, prevented Ang II-induced AAA formation in apoE^–/–^ mice by upregulating SIRT1 in VSMCs (123). Resveratrol (RSV), a natural activator of SIRT1, significantly alleviated Ang II-induced VSMCs senescence (124). A selective activator of SIRT1, SRT2104, was found to improve serum lipid metabolism parameters including total cholesterol, low-density lipoprotein and triglycerides in CVDs (125), and also attenuated serum pro-inflammatory cytokine levels and coagulation activation induced by intravenous LPS in healthy subjects (126). Senescent mechanisms and intervention measures for AAA patients based on SIRT1 are listed in Figure 3.

### Mitochondrial dysfunction-related senescence and abdominal aortic aneurysm

Mitochondrial dysfunction is an important cause of cellular senescence, which is different from senescence caused by genotoxic stress. Mitochondria oxidize NADH to NAD^+^ to maintain their normal function, but when mitochondria are dysfunctional, the ratio of NAD^+^ to NADH is out of whack, leading to the activation of adenosine monophosphate activated protein kinase (AMPK) and p53, which triggers mitochondrial dysfunction related senescence (miDAS) (127). Mitochondria are the main source of ROS, and mitochondrial dysfunction may contribute to abnormal ROS production (128). Specifically, elevated ROS levels impair the lifespan of vascular cells during cellular senescence and have been demonstrated in human brain aneurysms. Dysregulation of mitochondrial dynamics is also a key factor in cellular senescence, which is mainly regulated by fission and fusion processes and is closely related to cardiovascular diseases (129, 130). Fortunately, studies have confirmed that targeted inhibition of Drp1, a key protein of mitochondrial fission, and enhancement of Mfn1, a key protein of mitochondrial fusion, can inhibit the production of ROS and the senescence of VSMCs to maintain mitochondrial integrity, which is a potential treatment method to inhibit the formation and progression of AAA (131, 132). In addition, CR is a non-pharmacological intervention that regulates mitochondrial function to prevent AAA formation. Mechanistically, p53 knockout almost completely blocks the protective effect of CR by inhibiting the activity of cytochrome C oxidase assembly protein 2 (SCO2)-dependent mitochondrial complex IV. On the contrary, SCO2 overexpression restores the beneficial effect of CR on antagonizing Ang II-induced expression of AAA-related molecules and ROS production in VSMCs (133).

**FIGURE 4:**
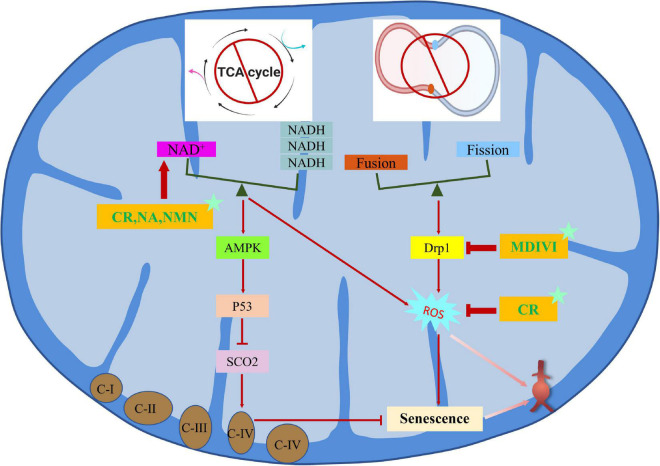
Regulation mechanism and intervention between mitochondrial dysfunction-related senescence and AAA.

MiDAS is characterized by imbalance of NAD^+^/NADH and mitochondrial fusion/fission. It is noteworthy that SIRT1 is an NAD^+^ dependent histone deacetylase, so SIRT1 is closely related to NAD^+^/NADH (108). The therapies that improve mitochondrial function by exogenous supplementation of their precursors, such as nicotinamide riboside (NR) and nicotinamide mononucleotide (NMN), have been demonstrated to be effective in the treatment of CVDs *in vivo* experiments (134, 135). The natural compound resveratrol not only acts as an activator of SIRT1, but also acts as an antioxidant, helping to remove excessive ROS and reducing mitochondrial damage to protect cellular senescence and AAA (136, 137). In addition, mdivi1, an inhibitor of Drp1, can also effectively maintain mitochondrial integrity and reduce vascular senescence (132, 138). Intervention targets and methods related to MiDAS for the occurrence and progression of AAA are listed in Figure 4.

### Autophagy-related senescence and abdominal aortic aneurysm

Autophagy plays an important role through a complex network of autophagy related proteins (ATG) involved in the induction and formation of autophagosomes (139). The key step of autophagosome evolution is to transform LC3-I into lipidized LC3-II (140). Cellular senescence under normal and pathological conditions is often closely related to the reduction of autophagy (141). Aortic autophagy decreases with age, leading to age-related dysfunction of endothelial cells (ECs) and arterial calcification (142). Defective autophagy has been shown to promote senescence in VSMCs. It was reported that a large amount of SQSTM1/p62 was accumulated in the VSMCs of ATG7 knockout mice, which accelerated the occurrence of SIPS, such as cell enlargement, nuclear hyperplasia, cell cycle arrest and increased SA-β-Gal activity (143). Importantly, trehalose or spermidine can slow age-related aortic pulse wave velocity and reduce the accumulation of oxidative stress and advanced glycation end-products (AGEs) in arterial wall (144). At present, there are few mechanisms about how autophagy regulates the senescence of aneurysm wall cells, but the veil behind autophagy will be revealed with the further research.

### Other

The nucleoli are sites where ribosomal DNA (rDNA) transcription (mediated by RNA polymerase I or Pol I) and ribosomal biogenesis occur. Studies have shown that inhibition of rDNA transcription in eukaryotic cells triggers a conserved cellular stress response called nucleolar stress (also known as ribosomal stress) (145, 146). Nucleolar stress induces activation of p53 pathway, thus promoting cellular senescence (147). In human AAA tissue, increased nucleolar stress in the medial cells is accompanied by localized DNA damage responses in the nucleolar septum. *In vitro*, nucleolar stress induces atypical DNA damage responses, mediating phosphorylation of p53 and senescence of VSMCs (148). However, clearance of rDNA transcription disturbance and nucleolar stress can contribute to recovery and prevention of AAA (148–150).

In addition, studies have found that both exogenous PM2.5 and endogenous intestinal microbiota metabolite trimethylamine *N*-oxide (TMAO) can mediate VSMCs senescence and promote the formation of AAA (151, 152). Other researchers found that treatment with soluble recombinant human ApoER2 or hydroxyurea, a DNA synthesis inhibitor, can inhibit PCSK9-induced VSMCs senescence (153). Meanwhile, a study have shown that inhibition of VSMCs senescence by targeting the MKL1/p38/MAPK pathway can be a potentially effective method for the treatment of AAA (154).

At the same time, a study found that excessive mechanical stress induces the continuous release of ADP and promotes VSMCs senescence through P2RY12-dependent Ras activation, which leads to excessive inflammation and degeneration and ultimately accelerates the formation and progression of thoracic aortic aneurysm (155). Another study reported that the reduced number and impaired function of circulating EPCs in patients with intracranial aneurysms may contribute to the pathophysiological process of aneurysm formation (156). In addition, a study has confirmed that cellular senescence is closely related to the formation process of retinal microaneurysms in the elderly (157).

## Conclusion

With the discovery of the physiological and pathological effects of senescence, many researchers have focused on the exploration and development of therapeutic methods targeting senescent cells. On the one hand, we have made good achievements in pre-clinical basic research, such as the research and development of senescent cell scavenger and SASP inhibitor(dasatinib + quercetin), which can effectively improve the continuous deterioration of senescence and even reverse senescence. Of course, on the other hand, we still need to do a lot of more difficult work, such as the search for specific markers of senescence cells, the heterogeneity and dynamics of senescence, the combined application of anti-senescence and pro-senescence therapy, and the precise change of SASP. However, as biological approaches and drug discovery technologies continue to advance, the underlying mechanisms that regulate senescence will come to light, offering promising transformational opportunities for the development of new treatments that minimize the harmful consequences of senescence and bring great benefits to elderly patients with AAA.

Although a large amount of evidence has been found to prove that cellular senescence is closely related to AAA, there are still some limitations in this paper. 1. Most of this paper discusses the evidence of VSMCs senescence in the vascular wall, but lacks the description of ECs, macrophages and other vascular cells. 2. The evidence chain between cellular senescence and AAA has not been fully elucidated in some of the included literature.

In this paper, we conclude that the reduction of SIRT1 molecular activity, on the one hand, leads to cell cycle arrest through p53/CDK2 signaling, and on the other hand, induces inflammation through the activation of NF-κB signaling pathway, both of which jointly promote aging and eventually form AAA. Therefore, designing drugs targeted SIRT1 may be effective in preventing AAA formation and progression.

## Author contributions

DW and SX devised the research plan. DW wrote the original draft. DW, XH, and LJ drew the picture. YJ, BJ, and SX modified and polished the manuscript. All authors contributed to the article and approved the submitted version.

## Abbreviations

AAA: abdominal aortic aneurysm
AMPK: adenosine monophosphate activated protein kinase
AGE: advanced glycation end-product
BET: bromine domain and extra-terminal
CDKI: cycle-dependent kinase inhibitor
C/EBP β: CCAAT/enhancer binding protein β
CVD: cardiovascular disease
DDR: DNA damage responses
EC: endothelial cell
EPC: endothelial progenitor cell
FA: fatty acid
JAK: janus kinase
LA: licorice chaltone A
MCP-1: monocyte chemotactic protein-1
MDM2: mouse double minute 2
miDAS: mitochondrial dysfunction related senescence
MSC: mesenchymal stem cell
NAD^+^: nicotinamide adenine dinucleotide
NF- κ B: nuclear factor kappa-B
NMN: nicotinamide mononucleotide
NR: nicotinamide riboside
rDNA: ribosomal DNA
ROS: reactive oxygen species
SIPS: stress-induced premature senescence
SAHF: senescence associated heterochromia lesions
SASP: senescence-associated secretory phenotype
SIRT1: silencing information regulator 2 related enzyme 1
STAT: signal transduction and transcription activator
TMAO: trimethylamine *N*-oxide
TVA: tricarboxylic acid
VSMC: vascular smooth muscle cell.
